# Remarks on Medical Reform

**Published:** 1812-11

**Authors:** 


### *To the Editors of the Medical and Physical Journal.*

GENTLEMEN,

BY inserting the following letter in your useful and widely extended Journal, you will oblige one of your readers.

Your Correspondent H. has, in your Journal for June last, entered the lists for his professional brethren with a degree of zeal and spirit which does him credit; but I differ widely from him in the conclusions he draws from the resolutions entered upon by the medical men of Kendal. I am very much inclined to think that the spark kindled at Kendal (to use his words) will not extend itself, nor do I suppose it ought to do so, for reasons which I shall by-and-by state: not that I suppose the country surgeon is adequately remunerated for the sacrifice of time, and the exertion of skill he daily makes in the service of his fellow creatures. To every one who gives the subject a moment's consideration, it must appear, that, were his fees three times as high as they are, he would not be overpaid.

But let us examine what is meant by the word Surgeon. By it we are to suppose a man completely qualified to practise medicine, surgery, and midwifery, for such is the duty the country surgeon is called upon to perform; but, comparatively speaking, how few of those men who usurp the name of Surgeon, in the country, are so qualified. It is from this circumstance, circumstance, most unquestionably, that the respectability of the profession is lessened; and, by consequence, that adequate remuneration does not accrue even to those who are, and conduct themselves as, surgeons.

It is a fact, which, I am sorry to say, I can bear witness to, whatever pain it gives me in so doing, that many of those men who call themselves surgeons in the country districts, are not so; that they are ill educated and ignorant, moreover dissipated and presumptuous.

It is more from the medical men in large towns being such as have enjoyed the advantages of liberal educations, and, by their habits, supporting that respectability due to their profession, that they are more adequately repaid for their attendance, than from the inhabitants in such towns being better able to make them such compensation.

A circumstance which tends greatly, or which is, perhaps, the sole cause of country towns being so much infected with those pretenders to the healthy art, is, a practice which at present prevails of men beginning to practise medicine under the assumed name of Surgeon, without any diploma, or ever having undergone any examination before competent judges, to prove that they were qualified to perform the important duties they may be called upon to do.

I have often been astonished at the presumption of men, who, without education, or, in fact, any qualification, to recommend them, (unless presumption and cunning can be considered such,) commence the practice of medicine; get upon their doors, in large capitals, SURGEON; and impose themselves upon the county as persons qualified to act the part of medical practitioners as well, nay, sometimes better, their impudence is so unbounded as to make them say, than the most learned surgeon in the place where they reside. This, gentlemen, is a circumstance which every country practitioner knows, and which every enlightened one must have had to lament at one time or other; for what can be more galling to the feelings of a man of sentiment, of education, and real professional skill, than to be ranked with such ignorant and illiterate persons. Those men are infinitely worse than professed *quacks*, for they are so in reality, disguised under the name of *surgeon*.

To me it appears extraordinary that the law should prevent men from following particular trades (that of a shoemaker for example) without not only being regularly brought up to it, but unless they are able to produce some proof of their ability to practise that trade; while any one can take upon him to practise the most important profession of medicine without being required to give such proof, can sport with, with, and, I am shocked to say, sometimes destroy, the life of a fellow creature with impunity, *quæ que ipse miserrima vidi.*

For the removal of this nuisance, an effort should communicate itself extensively, the whole medical population (properly so called) should exert its influence; in short, it is a duty which every humane man owes to society, to do his utmost to eradicate this evil, more baneful in its effects than is generally supposed.

A reform in this respect would, I apprehend, be more beneficial to society, as well as to surgeons, than possibly could result from exertions such as has been made by the medical men of Kendal: indeed the very nature of such proceedings sanctions the existence of the *illiterate* alluded to, and it was for this reason that I said the plan ought not to extend itself; but they do more, they place them upon the same level with the most learned and most worthy surgeon.

Let us, for example, take the medical men of Kendal. The gentleman who drew up the resolutions we are speaking of, I believe I have the honor of knowing personally, and I know him to be a man of science, of erudition, and real professional merit, and to whom I should consider three times the sum proposed scarcely adequate for his attendance, &c.; but, for aught I know, there may be *illiterate* persons in that town who practise medicine. Now, is it fair that a gentleman, such as I have described the above, should receive no more for his trouble, attendance, &c. &c. than them? besides the violence done to his feelings in being ranked with such.

It appears to me then, that the most effectual means of making the country practitioner better remunerated for his services, would be to raise the respectability of the profession to its due height, which is only to be effected by those who are meant to act in that capacity following a different, a very different, plan of study; for what confidence, or what reward, is due to a person, who, after a three years' apprenticeship, during which every intelligent medical man knows a great deal is not learned, even under the most skilful masters, goes to Edinburgh or London for the space of six, eight, or ten, months, then returns home and commences practitioner, without ever having been proved qualified; after which time he seldom looks into a book, except perhaps a translation of the Pharmacopœia, a Latin one many cannot translate!

I say, that if, instead of such a course of education, no man would begin to practise medicine until he was properly qualified so to do, until he had obtained his diploma from some respectable college; and, if those who could not give such proof of ability, were treated with that neglect which is due to ignorance and presumption; by these means, more would be done for those surgeons really deserving the confidence of the public, and an incalculable advantage result to society.

I will, at present, only further add, that I do not wish to insinuate that there are not men who, although not possessed of diplomas, are undeserving the confidence of the public; there are some such undoubtedly, and for my own part I am truly astonished that they can possess a moment's peace of mind, for reasons which my present limits will not allow me to notice. I have the honor to be, Gentlemen,

With the greatest respect,

Your most obedient Servant,

*Westmoreland, August* 1812. L——, Surgeon.

P.S. Is it legal for any one to style himself Surgeon without being possessed of a diploma? It is well known that a person may be prosecuted for assuming the title of Dr. without a degree;—why should Colleges of Surgeons not have the same privilege?

